# Recent Advances in Bipedal Walking Robots: Review of Gait, Drive, Sensors and Control Systems

**DOI:** 10.3390/s22124440

**Published:** 2022-06-12

**Authors:** Tadeusz Mikolajczyk, Emilia Mikołajewska, Hayder F. N. Al-Shuka, Tomasz Malinowski, Adam Kłodowski, Danil Yurievich Pimenov, Tomasz Paczkowski, Fuwen Hu, Khaled Giasin, Dariusz Mikołajewski, Marek Macko

**Affiliations:** 1 Department of Production Engineering, Bydgoszcz University of Science and Technology, Al. Prof. S. Kaliskiego 7, 85-796 Bydgoszcz, Poland; techniczny.tomasz@gmail.com (T.M.); tompacz@pbs.edu.pl (T.P.); 2 Department of Physiotherapy, LudwikRydygier Collegium Medicum in Bydgoszcz, Nicolaus Copernicus University, 87-100 Torun, Poland; e.mikolajewska@wp.pl; 3 Neurocognitive Laboratory, Centre for Modern Interdisciplinary Technologies, Nicolaus Copernicus University, 87-100 Torun, Poland; 4 Department of Aeronautical Engineering, Baghdad University, Baghdad 10001, Iraq; dr.hayder.f.n@coeng.uobaghdad.edu.iq; 5 School of Control Science and Engineering, Shandong University, Jinan 250100, China; 6 Laboratory of machine Design, Lappeenranta University of Technology, 53850 Lappeenranta, Finland; adam.klodowski@lut.fi; 7 Department of Automated Mechanical Engineering, South Ural State University, Lenin Prosp. 76, 454080 Chelyabinsk, Russia; 8 School of Mechanical and Material Engineering, North China University of Technology, Beijing 100144, China; hfw@ncut.edu.cn; 9 School of Mechanical and Design Engineering, University of Portsmouth, Portsmouth PO1 3DJ, UK; khaled.giasin@port.ac.uk; 10 Institute of Computer Science, Kazimierz Wielki University, 85-064 Bydgoszcz, Poland; dmikolaj@ukw.edu.pl; 11 Faculty of Mechatronics, Kazimierz Wielki University, 85-064 Bydgoszcz, Poland; mackomar@ukw.edu.pl

**Keywords:** robotics, bipedal locomotion, human gait, bird gait, synthetic-based biped gait, humanoid, sensors

## Abstract

Currently, there is an intensive development of bipedal walking robots. The most known solutions are based on the use of the principles of human gait created in nature during evolution. Modernbipedal robots are also based on the locomotion manners of birds. This review presents the current state of the art of bipedal walking robots based on natural bipedal movements (human and bird) as well as on innovative synthetic solutions. Firstly, an overview of the scientific analysis of human gait is provided as a basis for the design of bipedal robots. The full human gait cycle that consists of two main phases is analysed and the attention is paid to the problem of balance and stability, especially in the single support phase when the bipedal movement is unstable. The influences of passive or active gait on energy demand are also discussed. Most studies are explored based on the zero moment. Furthermore, a review of the knowledge on the specific locomotor characteristics of birds, whose kinematics are derived from dinosaurs and provide them with both walking and running abilities, is presented. Secondly, many types of bipedal robot solutions are reviewed, which include nature-inspired robots (human-like and birdlike robots) and innovative robots using new heuristic, synthetic ideas for locomotion. Totally 45 robotic solutions are gathered by thebibliographic search method. Atlas was mentioned as one of the most perfect human-like robots, while the birdlike robot cases were Cassie and Digit. Innovative robots are presented, such asslider robot without knees, robots with rotating feet (3 and 4 degrees of freedom), and the hybrid robot Leo, which can walk on surfaces and fly. In particular, the paper describes in detail the robots’ propulsion systems (electric, hydraulic), the structure of the lower limb (serial, parallel, mixed mechanisms), the types and structures of control and sensor systems, and the energy efficiency of the robots. Terrain roughness recognition systems using different sensor systems based on light detection and ranging or multiple cameras are introduced. A comparison of performance, control and sensor systems, drive systems, and achievements of known human-like and birdlike robots is provided. Thirdly, for the first time, the review comments on the future of bipedal robots in relation to the concepts of conventional (natural bipedal) and synthetic unconventional gait. We critically assess and compare prospective directions for further research that involve the development of navigation systems, artificial intelligence, collaboration with humans, areas for the development of bipedal robot applications in everyday life, therapy, and industry.

## 1. Introduction

Mobile robots of various locomotion mechanisms have revealed limited built-in autonomy that obtains information from both internal and external sensors for pre-planned and purpose-oriented locomotion [1,2,3]. Legged robots offer greater possibilities than wheeled and tracked robots in terms of working environments. Legged robots can move over regular and irregular terrain without any hardware modifications and have demonstrated exceptional mobility [4]. Generally, the two-legged robot mimics the way human moves. It is intended to undertake a variety of tasks including civilian and military activities in hazardous conditions, entertainment and education, and assistance for the elderly and the disabled. Owing to the smaller foot contact area with the ground and the smaller number of driving effector, bipedal walking robots’ total energy consumption may be lower in comparison to the multi-legged robots [5]. A historical overview of leg-driven robots and machines, as well as an introduction to the walking pattern and stability generators, are provided in the articles by Bekey GA [6], Raibert MH [7], and Al-Shuka [8,9], respectively. The information gathered by the robot sensors can be used for real-time analysis of the environment. Moreover, there are cognitive robots such as the intelligent robot companions, which may be helpful in the therapy of children with autism spectrum disorder (ASD), depression, or as an aid for the elderly [10,11,12,13]. Generally, the common challenges of biped robots include, but are not limited to, the following:

- Bipedal robots have unstable structures due to the passive joints located at the unilateral contact between the foot and the ground [14,15,16];
- One-sided contact of the foot with the ground and a complex configuration of the gait cycle bring about the highly non-lineartrajectory of the bipedal robot [14,17];
- Bipedal robots have multiple degrees of freedom (DOFs). Most researchers use simplified models to reach a trade-off between simplicity and the dexterity [18];
- Bipedal robots are most often designed to interact with unknown environments and are expected to achieve a high level of autonomy [19,20];
- Simulation is required as a part of many control strategies for bipedal walking [19,21].

These topics are related to advanced mechanics, control theory, electronics, artificial intelligence (AI), and the knowledge of human anatomy. Obviously, the research and development of bipedal walking robots are truly interdisciplinary. To solve these problems, the close cooperation of research teams from various fields is required. As far as we know, the first walking robot is described in theIlliad, by Homer. A wooden walking device named Mu Niu Liu Ma (in Chinese) was designed in the year 231. Modern studies on humanoid robots began in the early 1960s with artificial hands and arms—for supporting the physical work of men. In 1969, Vukabratovic et al. [22,23] developed several original self-propelled exoskeletons to help paraplegics. However, the most well-known humanoid robot is Asimo, created by Honda in the year 2000, based on prototype E0 (1986) [24].

Review articles on bipedal robots can be found in the scientific literature [25,26,27,28,29,30]. Wahde et al. [25] presented the research progress of biped and humanoid robotics in the year 2002. The authors described both commercial and research projects showing biologically inspired biped robots. Bezerra et al. [26] presented a review of the main types of biped robots that developed until the year 2004. Silva et al. [4] provided a review in the field of optimization methods for the construction and manners of movement of walking robots, which were characterized by greater energy consumption, as compared to robots on wheels [27]. Ficht et al. [28] investigated an important aspect of the development of modern walking robots—a humanoid robot with a fully 3D-printed structure supported by off-the-shelf components. This method significantly reduces the implementation cost and allows modifications of the structure. Ye et al. [29] presented a brief overview of the methods thatenable stable walking and running in bipedal robots. Modern methods significantly improve the endurance and adaptability of robots. They can traverse unknown terrain with a variability of the ground exceeding 20% of the length of the legs. Bipedal robots can regain balance after a sudden push, not only in a stationary state but also while in motion. However, running is still a problem for robots.Ficht et al. [30] provided an overview on the most advanced solutions for two-legged robots. The article presented the applied kinematic structure of the leg drive of modern humanoid robots, and the directions of development of bipedal robot control systems. Particular attention was paid to the technological aspects, emphasizing the role of 3D printing in creating new solutions. Gupta and Kumar [31] have made a good review paper concerning design models and control strategies for motion and running of underactuated biped robots.There are interesting works on the analysis of the dynamic walking of biped robots, for example, see the works of Westervelt et al. [32] and Sadati et al. [33].

Regarding the broad topic of the bipedal walking robots, there is a twofold direction of research, distinct from each other: (1) the progressive research about bipedal walking robots, and (2) review studies (surveys of the literature) on the various research and to the results obtained so far—a survey of the literature. In the present work, we accomplished this in the second category. We are conscious that the current paper, despite more than 160 references cited, may address this paper more deeply—thus, it may be regarded as introductory for further deeper and narrower studies in their focus. We hope it will inspire researchers, engineers, and clinicians for further interdisciplinary studies paying attention to their various detailed aspects of the very important issue of robotic bipedal walking, e.g., low limbs exoskeletons, also controlled using a brain-computer interface as half body neuroprosthesis.

The focus of this article is to survey the challenges that relate to the theory of human and bird, design, drive, and control systems of bipedal robots. It considers the solutions based on the natural biped motion (human-like and bird-like) and also synthetic, heuristic solutions of bipedal walking machines. Section 2 presents the main biped gait issues of humanoids and birds. Section 3 focuses on the implementation of self-developed bipedal walking robots based onnatural gait patterns and syntheticsolutions. In Section 4, drive and control methodology of biped walking robots, the sensing methods to support their two-legged movement, and also energy efficiency are discussed. Section 5 shows the prospect of bipedal walking robots and their potentials. Finally, the summary and conclusions are presented in Section 6.

## 2. Bipedal Walking Mechanism

### 2.1. Bipedal Walking of Human

Walking requires integrated and coordinated activities of thenervous, muscular, and skeletal systems, which control and enable the gait, as well as provide the closed-loop feedback for reactive balancing and stability control. The main theories to explain the bipedal walking are listed below [5,8,9]:

- The evolution theory;
- Theory of minimizing energy consumption;
- The theory of maturation (grow from childhood to adulthood);
- Central pattern generator theory;
- The theory of bipedal locomotion as a result of the two cooperating mechanisms;
- The theory of bipedal robots (gait can be generated by stability and online feedback control [7].

Each of the aforementioned theories has some supports, but none of them comprehensively explains all possible physiological and pathological mechanisms. Researchers have attempted to extract the bipedal robot design inspirations from their biological counterparts, but there are still many problems regarding the gait complexity of different levels:

- The general view: the stable, controlled bipedal gait in various environments and during fulfilling various meaningful tasks (including cognitive tasks);
- Combination and transition between thedifferent modes of bipedal locomotion: walking and running without falling [34];
- The high-level control of walking: cooperation of trajectory planning algorithms and central pattern generators [35];
- The high-and low-level signal processing: translation of ground contact into signals activating particular joints and muscles [36,37];
- The adaptive control layer that considers dynamic stability, detecting ground movement or slippery surfaces during walking.

Many of the above-mentioned problems are possible to solve based on the theory of nonlinear dynamical systems (stability/equilibrium analysis, cycle properties, multifractal analysis) [35]. However, no one-size-fits-all criterion ensures the equilibrium of bipedal robots. 

### 2.2. Application of Bipedal Gait of Humans for Biped Robots

A better understanding of the bipedal walking robots comes from the analysis of human walking behaviour adaptation strategies that are based on interlimb and intralimb coordination. Goswami [38] analysed a biped robot’s postural stability and foot rotation. Furthermore, analyses of passive walking with knees were presented in [39]. Early studies of these mechanisms were based on a split-belt treadmill. A two-tier bipedal walking control model was described by Fujiki et al. [40]. Its main principles were based on the following:

- Independent neural control of parameters during gait;
- The coupling of the metatarsal to the medullary regulation;
- The ability to adapt and remember the new inter-acting patterns;
- Involvement of the cerebellum in generating substantive commands based on foot signals;
- The interaction of the cerebellum with the brainstem is a key control for interlimb coordination in slow walking [41,42,43].

Gait pattern is generalized to natural walking even if the vision is removed [44]. This has been achieved thanks to evolution, experience, training, and trial-and-error practice [45].

### 2.3. Concepts of Bipedal Walking Robots Based on Human Walking

Humans and animals have matchless mobility abilities due to their versatile mechanical reconfiguration capabilities. The features of animal and human locomotion systems are analysed [46,47]. During the movement, humans use about 20 DOFs from all the 300 DOFs of the musculoskeletal system [18,48,49]. Based on this observation, the following important observations from previous literature are summarized:

- The majority of researchers try to adapt the idea of human walking using simplified models [9]. Among possible choices there are 2-link model [8,50], 3-link model [51], 5-link model [17,52,53], 30-DOFs humanoid [54], etc.
- The complete gait cycle of human walking consists of two main successive phases: the double support phase (DSP, 20%) and the single support phase (SSP, 80%) with intermediate sub-phases (toe-off, forward swing, and heel strike) [54]. DSP results in a closed chain mechanism, while the SSP starts when one of the feet begins the forward swing phase [55].
- Balance and stability problem is important especially during the SSP when bipedal motion is unstab [56]. In general, there are two kinds of stability criteria: static and dynamic. Static stability depends on the vertical projection of the centre of mass on the support surface [57] and allows to simplify the design of bipedal robots considerably and open-loop balancing can be assumed with large enough feet [58]. For dynamic stability, the following methods are usually considered: zero moment point (ZMP) [57], centroidal angular momentum [59], footstep-based criteria [60], and periodicity-based gait [61,62,63,64]. Further details can be also found in previous literature [6,9,65].

**Remark** **1.** *Some researchers consider the ZMP-based locomotion as static motion as long as the walking is slow in comparison with motion of periodicity-based stability. In effect, most passive walkers lie within the category of periodicity-based motion. The passive walkers use minimum actuators for motion on a plane surface or even without actuators but with an inclined surface for walking. In contrast to active walkers that use full actuation or over actuation strategy for walking, most of them are designed based on the ZMP, see [31,66] for more details.*

**Remark** **2.** *The human walks using muscles and nerves as actuators and controller elements, respectively. Without the muscles and nerves, the human behaves as a passive walker [64]. Consequently, the bipedal robot can be modelled as an inverted pendulum with passive dynamic walking that exploits dynamics only, e.g., McGeer’s passive walker [67,68]. In view of the above, bipedal robots have been known to exhibit complex behaviour like chaos and bifurcations with hybrid algebraic-differential equations [64,69]. Simple models were selected to investigate chaos and bifurcations phenomenon such as compass-gait biped [70], point-feet straight legged robot [71], semi-passive biped actuated in torso [72], an underactuated bipedal robot with constant torque being applied on the support leg [73], a 3D passive walker [74], and rimless wheel models [75]. On the other hand, thePoincaré map is a powerful tool to investigate the stability of passive dynamic walkers; however, difficult computations arise in solution of complex biped systems. Therefore, Zengui et al. [69] proposed time linearization of the hybrid bipedal system with a state feedback controller to stabilize the linearized Poincaré map. In general, two basic methods are available for controlling chaos [76]: the Ott–Grebogi–Yorke method and the delayed feedback control method, see [64] and the references therein for more details.*

One of the important issues of biped locomotion is the generation of the optimal trajectory that provides the stability while avoiding collisions with obstacles. In practice, several algorithms can be combined to generate bipedal locomotion patterns [6]:

- The learning process (requires intelligence);
- A considerable level of ability to adapt to different conditions or to solve tasks of different obstacles in the terrain;
- Under certain conditions (e.g., long-distance walking), optimal movement to reduce walking energy consumption.

Most designers and scientists proposed control systems for bipedal robots based on predefined trajectories. Methods used for online ZMP compensation can be based on preview control [77], model predictive control [65,78,79,80,81,82], or AI [83,84]. In contrast, modern systems, based on artificial intelligence, can produce sound results without direct modelling of the phenomena [85], although there are different approaches adopted to generate balanced/stabilized walking patterns. Hayder A.S. et al. [9] present the classification of gait generation approach based on model, biological mechanisms, and natural dynamics [19,52,58,86].

### 2.4. Selected Issues of Bird Gait

In nature, several animals have been known to move perfectly on two legs such as dinosaurs. Thulborn [87] gave the known relationships derived from mammals such as speed, gait, and body size to determine the gaits and theoretical maximum speeds of dinosaurs. He concluded that smaller bipedal dinosaurs ran at speeds of up to 35 or 40 km/h, “ostrich dinosaurs” 35–40 km/h, and up to 60 km/h. Larger bipedal dinosaurs were walking or slowly trotting with speeds in the range of 15 to 20 km/h. According to the theory of evolution, birds are one of the closest successors of the dinosaurs—they also move on two legs independently of if they can fly or not. Alexander [88] analysed the gaits of different animals (reptiles, birds, and mammals). He concluded that mammals move similarly when the Froude number is considered, as shown in Equation (1):

(1) $$F=\frac{u^{2}}{\mathrm{gh}}$$

where: u is the speed, g denotes the acceleration, and h stands for the height of the hip.

In another paper [89], the author observed that, in addition to humans and birds utilizing bipedal walking mechanisms, cockroaches can also run in bipedal mode. Many birds walk and run with knees bent, back and femurs in a position near to horizontal. The author showed the differences between the two-legged movement of humans and birds. In humans, two peaks of force can be observed during walking, and a single peak when running. Two peaks of force similar to that of human walking are also observed in birds. Human walking is energy efficient, but human running is rather expensive. For birds, bipedal locomotion is economical for wading birds, and expensive for geese and penguins. Abourachid et al. [90] presented a short review of the biological bipeds to explain the differences between the body structures of humans and birds. The authors proposed a bird model scaled up to the same mass and height of the centre of mass as a humanoid model. The results of such simulation showed the advantage of the bird model in comparison with the humanoid model. Results have also confirmed the possibility of decomposition of the bird’s system on the trunk and thighs as one part, and leg as a second part. The authors noted that the movement of birds may be a good inspiration for building walking robots. Daley et al. [91] observed that birds are ecologically diverse and span a large range of body size and limb proportions, yet they all use their hind limbs for bipedal terrestrial locomotion. The authors also indicated that birds use different strategies depending on terrain such as:

- Independent control of the angular movement and the length of the legs to ensure dynamic stability;
- Control of the speed of movement with positive feedback to ensure a constant load on the legs in uneven terrain;
- Adjusting the muscles to the load, which stabilizes the mechanical energy usage of the body;
- Complex regeneration strategies that allow changing the dynamics of the body, while regulating the load on the legs, which in turn minimizes the risk of falling.

The authors [92] analysed the scaling of the gait of bipedal birds related to the load and muscle power that results in adapting the frequency of the gait. The stability and agility of movement were also analysed. At dynamically similar speeds, small birds use relatively shorter stride lengths and higher stride frequencies as compared to large birds. Birds with long legs as compared to their mass, use longer strides and lower swing frequencies. Birds are the only living animals that can stand, walk, and run on two legs, just like a human. At the same time, the fastest birds (ostriches) are much faster than humans. The movement of birds is also very agile. This is due to the different structures of the kinematic system and muscles in comparison to the human gait. Leveraging biological patterns of bird movement is an inspiration for bipedal robot’s alternative [90] to humanoid robots that have been developed for many years. The construction of bird-inspired bipedal robots will be presented in the next section.

## 3. Designs of Biped Walking Robots

### 3.1. Overview of Bipedal Robots

To reveal the current state of the art, a search and review of the literature on bipedal robots published up to 31 December 2021 was conducted using the following keywords: “bipedal walking” and “bipedal robot”. The following electronic databases were searched: Scopus, Pubmed/Medline, PeDro, Cochrane, Pro-Quest as far as MeRoDa (Medical Robotic Database). After evaluation of publications according to their titles, abstracts, and full texts, a narrative review was performed. In a six-year window, all articles dealing with the walking robots that can be found on Google Scholar represent around 10,000 titles. The state of knowledge is based mainly on narrative reviews as well as on previous technical reports. The design of bipedal walking robots presented in Table 1 can be divided into solutions based on the following biological patterns:

- Human Biped Walking Robots (HBWR);
- Bird Biped Walking Robots (BBWR);
- Synthetic Biped Walking Robots (SBWR)—other solutions based on a heuristic, synthetic ideas.

Humanoid robots are bipedal walking devices built to resemble human-like locomotion. Do-It-Yourself bipeds are characterized by a simple kinematic chain design to reduce actuation costs and simplify the control system; such designs are designed towards simple manufacturing—often low-cost 3D printing or laser cutting of all the parts. Another typical feature of such designs is their open-hardware and open-source licensing.

### 3.2. Human Biped Walking Robots (HBWR)

Most of the solutions presented in Table 1 are designed by imitatingthe human walking mechanism. Two-legged robots mimicking human walking mechanisms are capable of passing over or avoiding obstacles; however, they are characterized by complex construction and control. For an efficient walking pattern, the bipedal robot needs 12 DOFs, as in the best solutions of bipedal walking robots, for example ASIMO [24]. There are attempts to simplify the kinematic structure of two-legged robots to 6 DOFs (Lim andYeap walking robot [127]), which results in a moderate reduction of movement capabilities. The robots presented up to this point are most often equipped with rotary or linear actuators electrically driven by DC servos or stepper motors. Boston Dynamics, in 2013, unveiled the prototype of the two-legged walking robot Atlas [101], powered by a hydraulic system. Then in 2016 [102], the company presented an excellent design with a perfectly refined hydraulic muscular system. This type of drive provides enormous strength and excellent dynamics. Initially, the controls were in development and the robot’s gait was wobbly. In 2019 [103], Boston Dynamics presented Atlas with improved software, which does not directly control the robot’s joints; instead, it uses pre-programmed relations between the robot’s features and its relation to environment interaction. The Atlas robot uses the whole body for balancing and performing acrobatic movements like jumping over obstacles or back-flips [103]. Undoubtedly, the solution presented by the Boston Dynamics company sets a completely new level in terms of the construction of walking robots. 

### 3.3. Bird-Based Biped Walking Robots (BBWR)

Recently, new solutions for two-legged walking robots areput forward using biological patterns that are not inspired by human gait, instead, they resemble birds walking mechanics. The examples of such walking robots are Cassie [118] and Digit [122]. We can see the difference between the structure of leg joints for human-based robots and Cassie or Digit robots that are similar to birds’ lower bodies. The key feature of those robots is excellent manoeuvrability and the possibility to move in difficult terrain. The Cassie robot was a protoplast for the Digit, which, as an evolution design, was equipped with arms and a head containing a LIDAR system for navigation [122]. The Cassie robot presents excellent walking and jogging capabilities; in addition, it exhibits good manoeuvrability due to the quick rotation [139]. These robots quickly reached a high level of design in comparison to the best solution of humanoid robots due to the advances in control systems of humanoid robots and using a unique drive design. 

### 3.4. Synthetic Bipedal Walking Robots (SBWR)

The last section of Table 1 presented synthetic walking designs. The designs utilize a cyclical leg shifting mechanism to maintain stability by ensuring the centre of gravity projection on the floor stays underneath the grounded feet. Table 1 presents two such solutions that combine the sliding motion to produce vertical leg movement when transferring the load from one leg to the other. Wang et al. [135,136] proposed an innovative design of a two-legged robot called SLIDER, which used a sliding joint in each leg to replace the knee–hip rotary motion that was used in nature for leg lifting [135,136].

The design prepared at the Imperial College of London [135] uses linear movements of the straight leg in the hip, replacing the effect of knee–hip combined rotation. The innovative aspect of the SLIDER design is the compact and lightweight mechanism of vertical hip movement. The proposed system of locomotion of the SLIDER robot uses only 5 DOFs per leg—totally giving 10 DOFs. Simulated robot motion animations are presented in [136]. 

**Figure 1:**
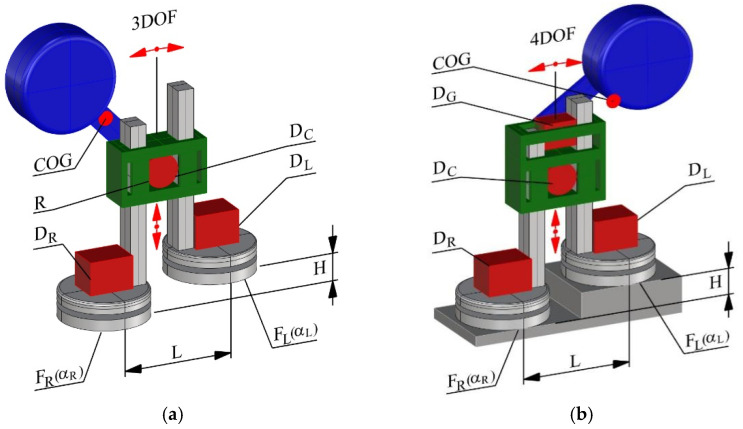
An innovative bipedal robot with swivel feet: D_R_, D_L_—drives of feet, F_R_, F_L_—swivel feet of robo, D_C_—drive of robots leg, COG—centre of gravity, L—distance between feet axis, H—vertical move of feet: (**a**) 3 DOF robot balancing mass moved using D_C_ drive; (**b**) 4 DOF robot balancing mass moving using D_G_—independent balancing drive.

One of the challenges in two-legged robots is the execution of twists and turnaround motions. The new innovative solution of walking mechanism—which is not based on human gait as most solutions of two-legged walking robots—was presented by Mikołajczyk et al. [131,132,133,134]. This new walking mechanism uses two parallel legs sliding in corps and is equipped with swivel feet as shown in Figure 1.

The 3 DOF robot is equipped with three drives (Figure 1a): central common drive—$D_{C}$ uses gear control of moving legs in the vertical direction, drives of swivel feet control: right foot—$D_{R}$ and left foot—$D_{L}$, respectively, on α_R_ and α_L_ angle. This way the robot is equipped with 3 DOFs, in which the 1st DOF is controlled by vertical simultaneous movements of the legs, and the remaining two DOFs are the rotations of the feet parallel to the ground surface (Figure 1a). For static stabilization, gear connection to balancing mass is used to move the centre of gravity—COG—from one foot to another. The movement of the mass in the simplest version is mechanically combined with the leg motions (Figure 1a). The sequence of a single robot step is as follows (the robot stands on two feet–starting from the left foot):

- The start of the central common drive ($D_{C}$) that rotates left moving the right leg concerning the left one, at the same time the mass moves and stabilizes the robot’s centre of gravity (see the example centre of gravity—COG—position) within the left foot’s footprint;
- The central driver ($D_{C}$) stops;
- Left foot ($D_{L}$) swivels motor is started to rotate the robot around the left foot by α_L_ angle;
- Left foot drive ($D_{L}$) is stopped when the final angular position is reached;
- Central common drive ($D_{C}$) starts to rotate right by φ angle lowering the right leg and at the same time the stabilizing mass moves to the upright position and the robot statically stabilizes on both feet.

Then the sequence repeats. When the $\;D_{C}$ drive moves on φ angle, legs move in the vertical direction by distance:

(2) H=2Rφ,

where: R is the radius of the gear and φ is the gear rotation angle.

During the walk, rotation of the foot is used for robot progression. The length of step S depends on the value of the rotation angle α ($\alpha_{R}$ or $\alpha_{L}$) and the distance between the legs L:

(3) $$S=2\mathrm{Lsin}\left(\frac{\alpha }{2}\right)$$

where: L is the distance between the legs, and α foot rotation angle.

The rotational degree of freedom in the foot enables changing the direction of locomotion, significantly exceeding the capabilities of conventional stepping robots.

A three-dimensional printed prototype of the 3 DOFs robot [131] was driven by three DC servos using the Pololu Maestro controller board. Thanks to the rotation of the foot, the robot is characterized by unprecedented agility among bipedal robots. Extending RotoFoot with the fourth degree of freedom (4 DOFs) at the torso, enabled the robot to climb stairs by improving control of the COG location using an independent balancing drive (D_G_) (see Figure 1b). A prepared 3D printed prototype of 4 DOF can climb stairs [134]. The new type of robotic synthetic drive can find different applications. For example, this robot can be used as an intelligent robot-companion or delivery robot. This type of walking robot seems to be particularly convenient to walk on flat surfaces; at the same time, the idea presented also allows for climbing stairs. 

A robot called LEONARDO (LEgsONboARDdrOne), or LEO for short, is a versatile design that enables two main modes of movement walking as well as flying [137]. This robot presents synchronized agile walking movements interspersed with flight manoeuvres. This allows it to perform manoeuvres that are difficult for traditional walking robots such as skating or walking on a rope. LEO consists of three main subsystems, namely a torso, a propeller drive system, and two legs with pointing feet. The robot legs are constructed of carbon fibre tubes and 3D printed carbon fibre reinforced joints with ball bearings. They constitute a parallel kinematic mechanism with brushless DC (BLDC) drives with gears located close to the torso, providing a compact form with reduced leg inertia. Both legs are symmetrical, and each leg has three servos for actuation. The first is located at the pelvis and moves the leg structure in the frontal plane of the LEO. The other two servo motors are located at the front and the back of the hips and drive the parallel leg mechanism. Thanks to applied solutions LEO weighs only 2.58 kg. Its height while walking is 75 cm. The solution uses high-friction urethane polygamy as a pointed foot with a load cell for ground contact. The robot uses sensors to detect the contact with the ground. 

LEO can then execute the walking phase using inverted pendulum control, but this is aided by the operation of the propellers. The robot’s construction allows it to change its configuration during the flight phase. LEO can operate completely autonomously with its onboard computers and sensor set; it also has other capabilities of moving all of which can be found in [138].

## 4. Drive and Control Systems of Biped Walking Robots

Modern walking robots are equipped with drive control systems and sensor systems to support both the operation of the robot’s mechanism and environmental recognition systems. The solutions applied in these systems for main bipedal walking robots are presented in this section.

### 4.1. Drive Systems

Ficht et al. [30] put forward the applied kinematic structure of the leg drive of modern bipedal robots. Both rotary and linear drives are summarized. He distinguished between five main causes of the structure of lower limb drives:

- Serial drive on-axis;
- Serial drive off-axis (requires gear);
- Parallel mechanism cranks-lever;
- Parallel mechanism, with a linear drive;
- Mixed serial/parallel mechanism.

Modern robots are driven by the use of electric drives, less often hydraulic ones [30]. Electric drive is used as:

- Harmonic drive (ASIMO [24], HRP-5P [121], Toro [105], TALOS [120];
- Cycloid driver (Cassie [118], Digit [122]);
- Muscle/tendons (Kengoro [116]);
- DC servo NimbRo-OP2 [119]);
- Serial elastic actuator SEA (Valkyrie [111], WALK-MAN [107]).

Hydraulic drives are used as:

- Servo-valves (Atlas robots [101]);
- Electro-hydrostatic actuator—EHA (Hydra [117]).

### 4.2. Control Systems

**Figure 2:**
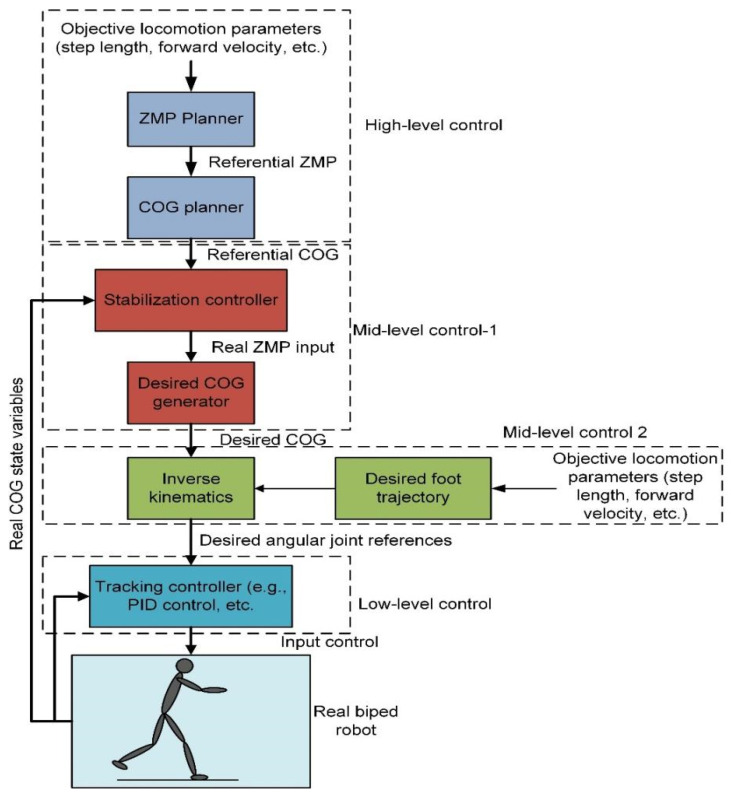
Multi-level control architecture with four control layers: High-level control, Mid-level control 1, Mid-level control 2, Tracking low-level control.

Four possible criteria are commonly used as the equilibrium/stabilization indicators for a bipedal robot: zero moment point (ZMP), point care map for limit-cycle walking, angular momentum-based criterion, and footstep-based criterion. As a result, the first two criteria are necessary to generate feasible biped motion, and the last two criteria are possible assistive indicators that can be used to recover the biped balance caused by external perturbation. There are explicit relationships between the ZMP, COG angular momentum, and the footstep, see [6] for more details. However, there are three problems related to the stabilization control of bipedal robots:

- First, how to generate the desired reference trajectory for a biped with high degrees of freedom.
- The second is how to guarantee (feasibly balance/stabilized) reference trajectory for the robot. This question is relevant if approximate models for trajectory planning are used.
- The third is how to precisely track the desired angular joint references considering the computational complexity of the high degrees of freedom of the biped. For example, Figure 2 suggests a general multi-level stabilization control for ZMP-based biped robot.

There is no prevailing approach. Simple models cover basic multisegmented ballistic and passive gait models. Ballistic bipedal walking robots can analyse internal signals such as energy consumption or the level of disturbances [140,141,142,143]. Simple mutually coupled Rayleigh oscillators for feedback control of a walking robot were described by de Pina et al. [144] and Luo et al. [145]. Zielińska described four coupled oscillators generating real-time outputs similar to human gait [57]. Chen et al. [146] presented sensor data fusion for the state of body estimation in stable walking using feedback control. Klein and Lewis provided a neuro robotic model based on Golgi tendon organs, and spiking neural networks [147]. Different feed forward strategies to recover from a trip or slip causing a fall due to response in muscle(s) excitation status were described by Forner-Cordero et al. [148]. Neural control models coupled with dynamical models driven by joint moments showed integrative properties of the neuromusculoskeletal systems within stable gait and individual muscle contribution to trunk support [149]. Even gait disorders associated with Guillan-Barré syndrome or spinal cord injury may be effectively reflected in walking assistant robots [150,151]. Based on the previous studies, it can be assumed that stable bipedal locomotion may be achieved by combining:

- Reflex-based control (e.g., artificial neural network) [152];
- Signals from local sensors;
- Simplification of bipedal robot kinematic construction;
- Adaptive compensation of small disturbances through controlling its dynamical properties [153].

For more details on control strategies of biped walking that are based on energy efficiency, see, e.g., [33,62,64]. Multi-level control architecture with four-level layers using different sensors is presented in Figure 2.

**High-level control.** The highest level of the inverted pendulum problem has to be solved using a model (IPM), see [6]. There are two essential problems at this control level:

- How to keep continuous COG state variables while changing the biped status/orientation? A modification to the IPM is required as discussed in [2].
- How to reduce the modelling error caused by the IPM inaccuracy? This can be answered by the mid-level control 1.

**Mid-level control 1**. It is responsible for a compensation of modelling error that results from the high-level control. Proportional–integral–derivative (PID) control or advanced control strategies, e.g., preview control can be used for regulation of the ZMP indirectly by tracking the referential COG. 

**Mid-level control 2**. It includes the inverse kinematics model of the robot and algorithm based on the calculation of the desired COG generated by the last level control and the referential foot trajectory. This control level can be avoided if task space coordinates-based dynamics are used rather than joint space-based dynamics. 

**Tracking low-level control** is based on the precise observation of the reference points of the joints defined by inverse kinematics. A simple PID or advanced control structure can be used in this control layer. This control is recommended to be distributed or decentralized for a high number of DOFs biped mechanisms to avoid computational problems. 

### 4.3. Sensor Systems

Position sensors on the motors provide data during locomotion that are key for both current state identification during the gait and biped robot further steps planning. Chen et al. [146] described a joint sensor system with a Kalman filter for the feedback control of the walking robot. 

The sensor system of biped robots was presented in [154,155,156]. Control of walking robots heavily depends on the progress in the field of mechatronics and the development of computer technology. Drive systems and advanced sensing and vision systems were the key developments enabling stable control of walking robots. There are new solutions for bipedal robots. The most advanced systems are developed in the USA and Japan, see Table 1 for details. For a biped robot to walk safely in complex environments, several sensor systems are required to sense/observe any obstacle or external objects. A good sensor system should be selected to recognize the correct foot placement while walking with different configurations. The sensor system can be subdivided into five categories:

- *Body orientation system.* To capture the trunk tilt of the biped, inertial measurement unit (IMU) sensors are commonly used, as they contain accelerometers and gyroscopes that through sensor fusion can be used for reliable orientation estimation. They are installed on the trunk in addition, some IMUs are placed on the feet to detect the feet inclination. Incremental, high-resolution encoders are often connected to joint motor shafts to measure joint positions and allow computation of positions and velocities. For detailed characteristics of these sensors, see the examples [154,155,156].
- *Foot sole sensor system* (Force sensors). The ground reaction forces play an important role in the stabilization of the biped mechanism and detecting ground stability. If these forces are outside the stability region, the foot may slip, and the biped robot might be not able to avoid a fall. Therefore, controlling these forces is necessary via confining the ground reaction forces to stay within the support foot/feet. This strategy meets the concept of ZMP. The ground reaction force wrench can be measured by placing four six-axes force/torque sensors on the foot sole.
- *Touch sensor system*. Some biped robots are designed to work in a home environment where there is a contact (touch) between the robot and human. Therefore, it is recommended to install the tactile sensor at specific locations to avoid trapping human hands/fingers in-between the robot joints. For example, [156] has used 19 tactile sensors placed inside the main elements of the robot. If these sensors are activated, then the biped robot attempts to release the joint forces.
- *Force sensing*. In the case of electric drives, monitoring of forces that robots can apply can be done using simple current sensing that can be part of the motor controller or external circuitry. For hydraulic actuation systems, pressure sensors installed on supply lines can be utilized to quantify force production.
- *Audio sensor system*. This sensor system is necessary for online communication with humans where a multi-microphone system is built. For example, the solution [156] installed seven audio sensors (microphones) on the head of the biped mechanism.
- *Visual sensor system*. Here, most typically, the head is equipped with a stereo camera-based vision system to identify objects and avoid obstacles, see [157] for more details on this topic.

### 4.4. Navigation Systems of Bipedal Robots in Uneven Terrain

Modern walking robots with environment recognition functions can move in unknown terrain [158,159,160,161,162]. They appeared as a response to the modern threats, where robots can replace humans in emergencies and harsh environments like fire, radiation (e.g., the Fukushima disaster), or in outer space [159]. Robot environment recognition systems are equipped with heads that include various sensors. The Light Detection and Ranging (LIDAR) system is used for real-world orientation. Visual sensors are effectively utilized for Visual Odometry (VO), and LIDAR sensors are used for LIDAR Odometry (LO) [162]. Unfortunately, such measurements commonly suffer from outliers in a dynamic environment, since frequently it is assumed that only the robot is in motion and the world is static. Moving objects in the environment need to be filtered out to provide reliable odometry.

Developed by Carnegie Melon University Multisense SL/SLB, a camera-based vision system was combined with a movable LIDAR sensor [159]. Such a system can be considered a fully functioning humanoid robot head as it allows the robot to “see” a very large scene around it without having to activate cameras or the neck. Several humanoid robots participating in the DARPA Robotics Challenge (DRC) [159] are equipped with Multisense SL/SLB, such as Atlas-DRC and Atlas-Unplugged [160], WALK-MAN [107]. Moreover, the birdlike robot Digit [122] from Agility Robotics uses a LIDAR sensor. 

Pieces of information from the camera-based system and LIDAR sensor are used by special processing software. The authors [161] present an algorithm for the probabilistic fusion of sensor data from different sensors (inertial, kinematic, and LIDAR) to produce a single consistent position estimate for a walking humanoid. Fallon et al. [160] describe the perception and planning algorithms that have allowed a humanoid robot to use only passive stereo imagery without LIDAR to safely plan footsteps to continuously walk over rough and uneven surfaces without stopping. Experimental results confirmed this idea. The robust Gaussian Error-State Kalman Filter for humanoid robot locomotion is presented in [163]. The introduced method automatically detects and rejects outliers without relying on any prior knowledge of measurement distributions or finely tuned thresholds.

### 4.5. Comparison of Known Modern Bipedal Robots Based on Human or Bird Walking 

In Table 2, some features of the most known modern bipedal walking robots whose design are based on human or bird’s gait are presented. Chosen robots are shown in order of the year of their first introduction. Certainly, robots are systematically developed. In this table summary of utilizing sensors, control systems, and type and number of drives are also given. Additionally, for some robots, their walking speed and load-carrying capacity for hands is also presented. All walking robots presented in Table 2 are equipped with sensors for monitoring the position of the joints. In addition, some are also equipped with torque or force sensors for joints. Most robots use IMU sensors for orientation in space. A few use the LIDAR system for this purpose. All humanoid robots are equipped with various types of vision systems. The Digit robot, which is rather a hybrid of a humanoid robot (torso with arms) and bird base walking platform, also utilizes LIDAR. The Cassie robot is not equipped with a camera. This model was mainly used to study walking and running functions based on bird ideas, while environment recognition and processing were not required in this project as the robot was controlled by an animator. The presented comparison shows that not only LIDAR can produce a good surrounding recognition function, but several robots also achieve excellent results in environment perception with the sole use of cameras. Apart from the Atlas robot, all other robots presented in Table 2 are equipped with various types of electric drives. However, the Atlas robot [96,97,98,160], which is equipped with a control system based on a LIDAR scanner and stereo vision and utilizes hydraulic motors for driving joints (Table 2) presents excellent possibilities in terms of precision and dynamics of movement, and the ability to move in difficult terrain.

An excellently tuned selection of sensors combined with state-of-the-art software provides distinctive opportunities for human interaction. It should be emphasized that the team of designers and programmers achieved these results in a relatively short time. Compared to the other robots, Atlas is distinguished by a much lower weight owing to the utilization of hydraulic drives that additionally provide excellent dynamics [103]. The performance of the bipedal walking robots using the bird’s movement pattern is also impressive: the low weight and the limited number of drives. Cassie [118] and Digit [122] robots are characterized by the original construction of the legs using the cycloid drive, which enables the use of very lightweight legs. It should also be emphasized that the slightly smaller NimbRo-OP2 [117] robot is made with the 3D printing technique, which ensures both low weight (due to the possibility of structure optimization) and a wide set of options for developing and improving the elements of the structure.

Advanced walking robots, which are based on a humanoid gait model, require a complex drive and control systems with many sensors. Moreover, a new type of knee-less walking mechanism [135,136] requires an advanced control system. It is a result of the number of DOFs. On the other hand, the developed synthetic walking mechanism for a bipedal walking robot with swivel feet [131,132,133,134] does not require a complicated control system due to the use of a small number of DOFs and its static stabilization method. The 3 DOFsRotoFoot robot requires three drives combined with three potentiometers-based position sensors for closed-loop control φ, $\alpha_{R}$ and $\alpha_{L}$α angles. The 4 DOFs robot, where the extra DOF is used for an independent mass movement (Figure 1b), enabled climbing stairs [134].

### 4.6. Energy Sources of Robots

Robots can be actuated in a number of ways; hydraulic, pneumatic, and electric drive systems are common solutions. In any case, the control and sensing systems require electricity for operation. This implies need for electrical supply in any way, which can be achieved by providing separate electric energy source for control or by utilizing energy conversion from the main energy source. Yang et al. [164] presents the summary of different energy sources for robotics. He divides all sources on:

- Energy storage, including batteries and capacitors/supercapacitors;
- Power generators—fuel cells, classical electromagnetic generators, and solar cells;
- Power harvesting (phototovoltaic, electrochemical, wireless, thermoelectric, etc.) and nanogenerators (micro-/nano-energy sources, self-powered sensors, and flexible transducers).

The main energy source for mobile robots is rechargeable batteries. For best performance, low weight, high current draw capability, and high capacity are required. Lowest cost per Wh can be obtained from lead-acid batteries, but they provide the lowest energy density per weight unit (35–40 Wh/kg) [165]. On the other end, LiFePO_4_ batteries are placed with the highest unit cost but also highest energy density per unit weight (90–160 Wh/kg) [166]. Some years ago, rechargeable battery technologies were used (Ni-Cd or Ni-MH–cell voltage 1.2 V), but the best are now Li-ion batteries with the highest energy density 100–265 Wh/kg, cells voltage 3.6 V, without memory effect and low self-discharge effect (1.5–2% per month) [167]. A problem with Li-ion batteries is the possibility to fire. Li-ion batteries for walking robots (1.1 kWh) produced by CEO [168] uses 4 ventilators for cooling of 98 cell (302.4 V). Detailed review for battery technology is provided in [169]. Li-ion batteries, however, have two main limitations—low energy capacity as compared to combustible fuels (petrol for example provides 13 kWh/kg, natural gas—15 kWh/kg, and hydrogen—34 kWh/kg), and relatively long charging time as compared to refuelling time. Compressed air can be stored with energy density in the range of 398 Wh/kg, which places it somewhere between batteries and combustion engines or fuel cells [170]. Hydrogen fuel cells are especially interesting, as electrical energy production is achieved without any moving components and almost noiselessly. Drawback of fuel cell technology is the weight of hydrogen tanks [171]. Diesel motor combined with hydraulic pumps and/or generator is a relatively simple solution for large robots; in smaller robots, such construction would have a significant vibration problem [172]. Fluid powered actuators provide advantage over electrical drives in terms of lower weight and high force, low speed characteristics that in electric drives are achieved using gearboxes that add to system weight and complexity. Fluid actuators characteristics are especially desired in walking robots.

Robots designed for short time operation can be powered by compressed air, electricity from supercapacitors, or batteries offering a relatively simple design. Electrical power storage is important in any case due to the control electronics and sensor systems utilized in robotics. Energy recovery or harvesting during operation requires an intermediate energy storage system that can accept large energy input in short time—for example in braking energy recovery. For this purpose, in case of electrical energy, supercapacitors are a good alternative; in case of fluid powered robots, pressure tanks serve the same function. Mechanical energy can also be stored for example in flywheels [173] or springs. Nevertheless, energy conversion between different systems is one of the sources of inefficiency of the drive train; therefore, it is foreseen that in the future most robots are going to utilize an energy source mix that supports actuation and control specific to the work environment of the robot and special application conditions. Currently, there is no ideal solution that would work in all applications. For indoor applications, combustion engines are not well suited due to noise and emissions, but recharging cycles can be planned with proper docking stations offering either pressurized gas, electricity, or hydrogen supply. For outdoor applications, and long operational time requirements, energy harvesting will be utilized as range extender, but the main power source will most likely become hydrogen or biofuels due to practical reasons and cost.

The robot size has a significant impact on energy source selection. Small scale with short duty cycles robots can be operated with just energy harvesting mechanisms and simple electric actuation systems that offer one energy source for all components is a clear winner, cost for dense electrical energy storage in small scale can also be justified in many applications. As the size of the robot increases and the duty cycle lengthens, denser energy storage with lower costs per unit energy is needed or fast energy refuelling either by battery swapping, tank refill, or quick charging mechanisms is required. The larger the size of the robot, the more benefit can be gained from energy source diversification and optimization for individual active robotic components.

For each robot, designers solved problems of energy source. DURUS, an 80 kg robot, used onboard 2.2 kWh lithium-polymer battery [103]. The BigDog robot, for instance, utilizes a small go-cart petrol motor (11 kW) for driving hydraulic pump and electric generator [174]; nevertheless, it was considered too noisy for military applications and development was turned towards fully electric drive. Pneumatic actuation can be achieved by integrating a compressor that is powered by electric or combustion motor, or by utilizing an air tank for compressed air storage. Robot Lucy is an example where external compressed air tanks or compressors are utilized [19].

Robots driven using hydraulic energy: PETMAN [89], ATLAS, and ATLAS DRC [102] used external sources of supply pressure. ATLAS unplugged [102] used source placed on board what increased mass from 152 kg to 182 kg. Actual version of ATLAS [103] based on improved design of hydraulic system has, similar to humans, a mass of 80 kg with a height of 151 cm.

Thangavelautham et al. [175] presents simulation results for a HOAP 2 humanoid robot that suggests a fuel cell powered hybrid power supply, superior to conventional batteries.

Possibilities to use new energy sources present a 88 mg micro robot RoBeetle [176] powered by catalytic combustion of methanol, whose specific energy is 5.6 kWh/kg. For comparison, the specific energy of an animal fat 10.6 kWh/kg and a good Li-ion battery is only 0.5 kWh/kg.

Intensive work is being carried out on the use of photovoltaics to power robots [177]. The relatively small surface area available for solar cells on the robot cannot convert enough energy to keep the power-hungry robot functioning, but with the technology, the cheaper perovskite cell achieves almost 24% efficiency, and specialist designs like the multi-junction (MJ) solar cell achieves as much as 40% efficiency [177]. Successful solutions of a solar robot, shaped like a sphere, are presented [178] and with wheels, Tertill [179] or Vitirover [180]. There is also a known small solar walking robot [181]. It is possible to use solar power to charge of rechargeable battery pack humanoid robots such as ATLAS [177].

The intensive development of batteries related to the automotive industry [182] allows us to expect that these sources will become the basic power supply for robots both in terms of propulsion power andin hybrid systems and control. The main directions are:

- Improve system of management of conventional Li-ion batteries;
- Improved design of Li-ion batteries by changing construction and composition lowers costs and improves performance (cobalt-free lithium-ion battery, mesoporous silicon microparticles and carbon nanotubes, lithium-sulphur batteries);
- New design and chemistry of batteries improve performance (vertically aligned carbon nanotube (VACNT), aluminium-air light battery);
- Design which improved time of charge (solid state lithium-ion batteries with sulphide superionic conductors);
- Structural batteries using carbon fibres as the negative electrode while the positive is a lithium iron phosphate; the latest battery has a stiffness of 25 GPa. It is possible to use it to design superlight electric vehicles and also walking robots.

This promises that future batteries in a short time will be lighter and will achieve much greater capacity, which will provide walking robots with a long operating time.

### 4.7. Energy Efficiency of the Walking Robot’s Motion

In some papers, the authors deal with the energy efficiency of a walking robot. This issue is very important due to the fact that robots are equipped with a limited amount of energy stored in the power source. This resource determines the range of the robot and depends on the efficiency of the drive system. The issue of energy efficiency of moving humans and animals was for the first time analysed by Trucker [183], who introduced the concept of the cost of transport (COT):

(4) $$\mathrm{COT}=\frac{P}{\mathrm{Wv}}$$

where: P—the power of move, W—weight, v—speed. Since the weight of the robot depends on the mass and gravitational parameter, the cost can be also presented as follows [127]:

(5) $$\mathrm{COT}=\frac{P}{\mathrm{Mgv}}$$

where: P—Power of robot, M—mass of the robot, v—speed, g—standard gravitation.

Kajita et al. [184] and Sakagami et al. [185] proposed specific resistance (SR) as an index for evaluating the energy efficiency of a mobile robot. It is based on the total energy consumption of d distance to the gravitational potential energy:

(6) $$\mathrm{SR}=\frac{E}{\mathrm{Mgd}}$$

where: E—Energy consumption, M—mass of the robot, d—distance of the move.

SR parameter is equivalent to the COT parameter. If the parameters of (4), (5), or (6) relations are all expressed in a consistent system of units, the quantity is dimensionless and has the same value in any system of units. Kashiri et al. presented an overview of principles for energy-efficient robot locomotion [186]. Based on references, it is possible to obtain some results of the COT or SR value of different robots and also to compare humans and four-legged robots. For MIT’s cheetah quadruped, which exploits electrical energy regeneration/recycling, the COT is about 0.5. A human is down around 0.2 [113], while Asimo (54 kg) exhibits COT = 2 for 1.5 m/s [185]. In the DARPA competition between robots, the best was SRI’s DURUS robot [113,187], which, with a fully charged battery, walked a distance of 2.05 km to run the battery dry in 2 h, 35 min, 43 s. The walking of the DURUS robot can be seen in the movie [114]. While walking, it used around 350 watts of power, giving it an average COT of about 1.5, which is better than the Atlas robot, which has a COT of 20 [113]. SRI estimates that in the future, it will be possible to obtain for the DURUS robot a COT under 1, and with its onboard 2.2 kWh lithium-polymer battery, the 80 kg robot should be able to walk 10 km [113].

SANDIA laboratory’s present special design of Walking Anthropomorphic Novelly Driven Efficient Robot for Emergency Response (WANDERER) [123]. It is possible to see the mechanism of the robot and its walking [124]. One of the best bipedal walking robots in terms of transportation cost is Cassie [118,188]. The new design of the Cassie robot (31 kg) has a low COT value, allowing Cassie to run for 6–8 h on a single charge. This robot based on the original design obtains excellent running results, completing 5 km in about 43 min [189]. When walking at 1.0m/s, using a total of 200 watts of power, while performing different locomotion behaviours such as squatting, the calculated COT was 0.7. For the LEO robot, v = 0.2 m/s COT = 108 was determined during walking [137]. When walking on the ground, LEO sucks down 544 watts, of which 445 watts go to the propellers and 99 watts are used by the electronics and legs [137]. LEO has a nominal walking speed of 20 cm/sec, and its total velocity relative to the ground can undulate significantly using intermittent near-ground flight. When flying, LEO’s power consumption almost doubles; it can also fly with speeds of 1–3 m/s. This high value is due to the high energy expenditure of the propeller drive system to stabilize the robot during gait. When flying at v = 1 m/s, COT = 48, and at v = 3 m/s, COT decreases to 15.5 [137].

## 5. Future of Bipedal Walking Robots

The future development of walking robots will depend on both the improvement and the appearance of new constructions, drives, and the use of various sensors for control movement systems as well as interactions with the environment and humans. Certainly, it will require the improvement of the operating software.

### 5.1. Limitations of Development of Walking Robots

The main limitations thatcan be deduced from the previous studies can be summarized as below:

- *Theoretical limitations*—there is a lack of comprehensive theories regarding the biomechanics, kinematics, and mechanisms of control/coordinating gait, ambulation, and clinical gait assessment; thus, it is hard to understand all aspects of bipedal gait, both physiological and pathological, and reflect them within the bipedal walking robot.
- *Technical limitations*—current technologies of bipedal walking robots are:
  ○ hard to assess due to limitations of computational models;
  ○ complicated design and control;
  ○ cost-effective only in several specialized applications, e.g., space exploration, military purposes, etc.
- *Cognitive and ethical limitations*—despite wide development (robots for the elderly, robotic toys for children, etc.), there is a need to increase common attention to the ethical limitations of using technology (including ICT and AI) for care interventions for people with limited self-awareness, insight, and orientation.

### 5.2. Achievements and Opportunities

There is a clear increase in open-source walking robotics projects in the last decade [130]. This indicates that the walking mechanisms will be widely used by students and even children, at first as toys, and later as scientific projects. Awareness of robotic capabilities in the general public is important before assistive robots and cooperation robots become easily available and widespread. Knowing from experience how robots perceive their surroundings and how to interact with them safely will lower psychological barriers that people have against new inventions. This in turn should lead to market-driven research on walking robotics directly applicable to common everyday tasks.

Bipedal walking robots may improve our understanding of the underlying neuronal control as far as biomechanical principles, mechanics, and muscle functioning of gait in individuals with gait impairments [147,149]. Modern bipedal robots are based on natural structures that move perfectly, both based on human gait patterns (ATLAS [103], Valkyrie [111], etc.) or birds’ template (Cassie [118], Digit [122]). Owing to the advanced sensory systems, precise, fast, and efficient drives can perfectly cope with moving in unknown terrain and respond to disturbances to maintain stability.

An important direction of development is the use of bipedal robot motion kinematics based on bird gait and run. The Cassie robot undertook testing in 2021 (with operator supervision) [189] and travelled 5 km in around 44 min at an average speed of 6.81 km/h. In one lap, the robot reached 7.74 km/h. The maximum speed achieved by this robot on long distances is more than the Atlas robot could achieve, as it reaches 5.4 km/h. Note, however, that the Atlas uses a hydraulic drive, while the Cassie robot is powered by a DC motor system. A bipedal walking robot became the basis for developing a neuromechanical simplified planar musculoskeletal model of human lower body biomechanics with a controller based on a dynamic artificial neural network with central pattern generators (CPGs) coupled with force and motion feedback to generate the appropriate muscle forces needed for walking. Separate neural networks generate the rhythm and create the gait pattern, especially stable in the sagittal plane without inertial sensors, a centralized posture controller or a walker in a manner similar to human walking (speed 0.850–1.289 m/s with a leg length of 0.84 m, also on 5° slopes without additional controller actions) [190]. An underwater bipedal walking soft robot based on a coconut octopus was designed and a machine vision algorithm was used to extract motion information for analysis—such a walking robot can achieve an average speed of 6.48 cm/s [191]. The bipedal walking robot has also become a test case for the use of shape memory alloy (SMA) springs as artificial leg muscles [192]. This is important for, among other things, locomotion rehabilitation and the development of assistive devices [193]. A better understanding of human movement increases the possibility of successfully combining humans and technology.

In addition to the construction of bipedal robots based on nature bipeds (humans or birds), the subject of research is unconventional solutions for bipedal robots:

- Innovative walking robots with swivel feet [131,132,133,134] with very simple kinematics provide excellent manoeuvrability (3 DOFs) and also the ability to climb stairs in the 4 DOFs version;
- A two-legged robot called SLIDER [135,136], which uses a sliding joint in each leg to replace the knee–hip rotary motion that is used in nature for leg lifting;
- The bipedal robot LEO with a versatile design that enables two main modes of movement by walking as well as flight [137,138].

Especially the latter solution seems to be useful due to the possibility of the robot moving both above the surface and in the air. Hence, the design and control of the multimodal robotic locomotion LEO allows for rope walking or skateboarding, which were previously challenges for bipedal robots [138].

### 5.3. Directions of Future Research

The aim of further work should be to combine the kinematics possibilities of bipedal robots and the possibility of safe interaction with humans. The authors of [158] presented a literature review on sensors of humanoid robot heads, especially focusing on aspects related to human–robot interaction. It has been observed that the robotic vision is the most widespread perception sensor system utilized in humanoid robots. This is mostly due to the amount of information that vision provides about the surroundings. The authors determined that there are two main types of humanoid robot heads and proposed a division into robot heads with non-expressive faces and robot heads with expressive faces. The latter is more human-like and, therefore, often perceived as more friendly. This will be the direction of further work along with the development of artificial intelligence, hybrid intelligence, and embodied intelligence of robots [194,195].

Taking into consideration wider perspective directions for further research should focus on:

- from a scientific point of view: knowledge sharing, including open-source solutions;
- from a technological point of view: on the development of robot navigation and artificial intelligence systems;
- from an organizational point of view: interdisciplinary collaboration among various research centres, virtual research teams, platforms for experiences, knowledge, and project sharing;
- from a clinical point of view: taking into consideration advanced applications of the aforementioned solutions in everyday therapy;
- from a societal and industrial point of view: dissemination of the knowledge and experiences, building social awareness concerning wider use of the bipedal robot walking in various areas of the daily life.

The most important research goals for the next several years may cover:

- high-quality studies to address research gaps within neural control and biomechanics of bipedal gait;
- the use of a highly dynamic hydraulic drive and the use of an innovative sensors system based on the LIDAR scanner system combined with an artificial vision system using stereo cameras set a completely new level in the field of robot’s world analysis;
- space exploration plans will mostly benefit from the development of autonomous walking robotics [194];
- a very important direction of the development of the bipedal walking robots mechanism will be further efforts to find solutions with high energy efficiency (low COT value). The goal is to approach and maybe even overcome the limit COT = 0.2 defined for some human walking conditions. This will allow increasing the range of robots with limited battery capacity.

### 5.4. Main Direction of Walking Robot Applications

The work carried out in the field of robot kinematics has produced valuable results indicating the possibilities of developing robot applications in various fields. Now walking robots are able to move both in urban terrain with unified flat wall and floor surfaces and stairs. Today’s robots are able to move in terrain with unfamiliar configurations. The development of energy sources and the improvement of energy efficiency point to the possibility of covering considerable distances. Some robots equipped with hands and touch sensors and equipped with a vision and recognition system can manipulate objects using cooperation of the upper limbs (Atlas [103], Digit [112]). Robots are also capable of using tools. In parallel with the development of the design and kinematics of robots, the development of artificial intelligence of robots is progressing. These developments are taking place at a pace beyond recent imaginings. Today’s robots can soon replace humans in dangerous and tedious tasks. Robots are being developed for tasks in space (Valkyrie [111]). Work is being carried out also on the use of walking robots for military purposes. The use of robots is likely to be driven by the need to increase productivity and save the environment.

Additionally, research on bipedal walking robots contributes to the scientific understanding of human movement, including the transition from walking to running [195]. The development of assisted locomotion with the use of exoskeletons may eventually make it possible to do away with wheelchairs or ramps, and disabled people will be able to enter places where fully able-bodied people enter. Bipedal locomotion and upright body position are also necessary for proper functioning of the human body (e.g., bladder and bowel function—certain muscles have to tense up and to relax).

Research into bipedal walking robots, both towards improving kinematic capabilities and developing artificial intelligence, could spur the creation of further well-designed assistive technologies:

- Solving problems in uncertain environments and assistance in hazardous places—firefighter support, zones with radioactive contamination, extreme temperatures, explosion risk zones, mines, outer space, etc.
- In the future, intelligent bipedal robots can be used in factories to serve as a replacement or a collaborator for human workers;in services for jobs that require taking on strenuous, uncomfortable positions or low-paying jobs that humans do not want to do, interactive robots may open new chances toward human–robot relationships, social awareness, activity monitoring, activity eliciting, and learning.
- Artificial environments such as virtual reality and augmented reality and brain–computer interface (BCI)may be more advanced alternatives for the traditional human–robot interface, providing multimodal interaction comparable to inter-human communication.
- Mobile technologies open new possibilities of remote control, e.g., for children/elderly safety and telemedicine/telerehabilitation purposes, as well as a general condition, sport, and leisure activities.
- Application of intelligent robots in space missions, both on their own and in supporting people, which may require the ability to walk.
- In conclusion, it should be stated that we are approaching the moment when robots in a much more perfect form than so far will enter our lives on two legs equipped with human-friendly artificial intelligence.

## 6. Conclusions

The presented survey of the literature as well as the previous work of the authors allows the formulation of the following conclusions:

- The artificial gait solutions of legged robots are based on the essence of the biological standard that has been created in the natural process of evolution through the development of joint muscles and the excellent biological control system;
- Gait inspirations for versatile application of walking robots can be gained from birds, insects, or other animals, not popularly used in walking research currently;
- Characterizing the essence of human gait and achieving it inmechatronics, drives, and control systems enabled the creation of solutions imitating this way of locomotion;
- The artificial gait systems based on a human biological pattern are built with varying degrees of complexity from 12–30 DOFs (close to human kinematics) to 6 DOFs or less (extremely simplified);
- Models imitating human gait mainly use electric drives with various motor types; in addition, especially recently developed walkers provide excellent performance using hydraulic drives or artificial muscles as in the Atlas robot;
- The new advanced design of bipedal robots based on natural patterns are Cassie and Digit, which use the pattern of bird gait. These robots use, with success, electric drives and are the fastest bipedal walking robots nowadays;
- The newly proposed solution based on the unique (synthetic) idea is a walking robot without a knee (10 DOFs);
- The bipedal innovative walking mechanism with excellent manoeuvrability equipped with two legs sliding in body with swivel feet allow to move on flat terrain at 3 DOFs, and at 4 DOFs, also climbing stairs becomes possible;
- An interesting design of walking robots is the robot Leo with a versatile drive, which can gait on two legs as a bipedal robot and fly as a drone;
- The conventional sensor system of advanced solution biped walking robots needs five categories of sensors systems of body orientation, foot sole, force, touch, vision, and audio.These kinds of sensors need both to solve the problem of moving robots by walking and communication of robots with the environment;
- Modern bipedal walking robots allow movement on flat surfaces in the field, the best solutions of a bipedal robot equipped with visual and LIDAR sensors can be used in uneven terrain to solve problems in conditions dangerous for humans—fire hazards, radioactive contamination hazards, or in outer space;
- Advanced walking robots that are based on natural biped gait need complicated drive and control systems with many sensors. This is related to the stabilization of the inverted pendulum system created by the legs. This problem does not exist in the presented biped (3 DOFs or 4 DOFs) walking robots with swivel feet;
- Combining the possibilities of building bipedal walking robots and equipping them with AI and human communication systems such as speech synthesis and recognition, and affective computing systems open new applications for these robots;
- The running time of the robots depends both on the performance of the power supply system but also on the COT parameter, which reaches for the DURUS robot a value of 1.5, and for the CASSIE robot COT = 0.7, when for humans the observed value is COT = 0.2;
- Walking robots use both hydraulic drives (ATLAS), which provide the greatest dynamics, and electric drives (DURUS, CASSIE, etc.). Rapid development of powerful batteries suitable for fast recharging is foreseen.

Modern robots have reached an excellent level of construction design, and drive systems that are capable to mimic perfectly natural walking, including automated locomotion in unknown terrain. It is especially important that the robots should become user-friendly and would not be in any way dangerous. The military applications of biped robotics are somewhat disturbing and might create a physical barrier to introduce biped robots into civil applications.

Future research will focus on the development of robot navigation and intelligence systems, as well as the interfaces for communication and collaboration with humans.

Taking into consideration a wider perspective, directions for further research should focus on:

- from a scientific point of view: knowledge sharing, including open-source solutions;
- from a technological point of view: on the development of robot navigation and artificial intelligence systems;
- from an organizational point of view: interdisciplinary collaboration among various research centres, virtual research teams, platforms for experiences, knowledge, and project sharing;
- from a clinical point of view: taking into consideration advanced applications of the aforementioned solutions in everyday therapy;
- from a societal and industrial point of view: dissemination of the knowledge and experiences, building social awareness concerning wider use of the bipedal robot walking in various areas of the daily life.

As authors represent nine independent research centres, each centre works on its project to solve part of the larger problem. We have developed collaboration mechanisms for remote group work utilizing online meeting platforms and cloud document sharing to facilitate long-distance teamwork. Information exchange and synchronization in terms of research funding applications are one of the keys to organizing distributed expert groups for multidisciplinary problems.

## Figures and Tables

**Table 1:** Summary of previous bipedal walking robot projects.

No.	Topic	Years	Type	Remarks
Experimental biped robots				
1.	WL-1 [93]	1966–1967	HBWR	Artificial lower limb. The base for further studies on bipedal robots.
2.	WL-3 [93]	1968–1969	HBWR	Bipedal walking device Master/Slave: walking, sitting, and standing device.
3.	WAP-1 [93]	1969	HBWR	A bipedal walking robot with artificial rubber muscles, pre-programmed gait sequence.
4.	WAP-2 [93]	1970	HBWR	A bipedal walking robot with effectors, automated posture adjustment thanks to feet sensors.
5.	WAP-3 [93]	1971	HBWR	First bipedal walking robot able to climb the stairs.
6.	WL-5 [93]	1970–1972	HBWR	Heavy bipedal walking robot with flexiblehips.
7.	WL-9DR [93]	1980–1982	HBWR	Quasi-dynamical robot. One step in every 10 s.
8.	WL-10, 10R [93]	1982–1983	HBWR	One step in every 4.4 s, possibility of turnaround.
9.	WL-10RD [93]	1984	HBWR	Dynamically stable robot. One step in every 1.3 s.
**Walking robots based on natural bipeds**				
10.	ASIMO [24]	1986	HBWR	Interactive robot. Gait velocity up to 5.95 km/h.
11.	HRP-2 [94]	2002	HBWR	Lifting objects, moving in unknown terrain.
12.	iCub [95]	2004	HBWR	Open-source robotics humanoid robot for research of human cognition and artificial intelligence.
13.	NAO [96]	2007	HBWR	Small walking robot for educational tasks.
14.	HRP-4C [97]	2009	HBWR	Female robot with a realistic face. Movement-based on captured human motion.
15.	HRP-4 [98]	2010	HBWR	Can collaborate with humans, exhibits human-like gait.
16.	PETMAN [99]	2011	HBWR	Protection Ensemble Test Mannequin.
17.	REEM-C [100]	2013	HBWR	Human–robot interaction.
18.	ATLAS [101,102,103]	2013–2020	HBWR	Search and rescue tasks, very dynamic, walking in uneven terrain, running, jumping capabilities.
19.	Robonaut 2 [104]	2014	HBWR	NASA robots get special legs with manipulation functions.
20.	TORO [105]	2014	HBWR	TORO is a humanoid robot controlled by torque used to study bipedal walking and autonomous manipulation.
21.	ATRIAS [106]	2015	BBWR	Bipedal robot inspired by bird gait kinematics.
22.	WALKMAN [107]	2015	HBWR	Rich sensory system control of loads and thermal sensing/fatigue of actuators and electronics.
23.	CHIMP [108]	2015	HBWR	Carnegie Melon University robot for rescue task.
24.	THORMANG [109,110]	2015	HBWR	Open-source advanced walking robot with the possibility to change to the wheeled platform.
25.	Valkyrie [111]	2015	HBWR	NASA’s Most Advanced Space Humanoid Robot.
26.	DRC-Hubo+ [112]	2015	HBWR	This robot can use tools, open doors, drive a vehicle, and transform into a wheeled robot.
27.	DURUS [113,114]	2015	HBWR	SRI’s robot with high energetic efficiency.
28.	HBS-1 [115]	2016	HBWR	Child size walking robots for different tasks.
29.	Kenogro [116]	2016	HBWR	Kenogro was equipped with body skeletal structure driven by muscle.
30.	Hydra [117]	2016	HBWR	Hydra uses electro-hydrostatic actuators (EHAs) with its own pump. It combines the advantages of hydraulic and electric drives.
31.	Cassie [118]	2016	BBWR	Dynamic robots walk and run as the animal (bird).
32.	NimbRo-OP2 [119]	2017	HBWR	Adult-sized open-source, low cost, a 3D printable humanoid robot.
33.	TALOS [120]	2017	HBWR	TALOS is humanoid, which can walk on uneven terrain, and perform tasks both in research and industrial environments (can operate power tools and lift 6 kg in each hand).
34.	HRP-5P [121]	2018	HBWR	A humanoid robot that can use a power tool and manipulate large objects.
35.	Digit [122]	2019	BBWR	Robots with many sensors based on Cassie kinematic for dynamical running in difficult environments, can do advanced tasks.
36.	WANDERRER [123,124]	2020	HBWR	Walking robot with an innovative mechanism for high energy performance and endurance.
**Do-It-Yourself bipedal walking robots**				
37.	DARwIn-OP, DARwIn-OP2 [125]	2011	HBWR	Dynamic Anthropomorphic Robot with Intelligence—Open Platform Robot humanoid kit.
38.	Low-cost 3D Printed Humanoid Robot		HBWR	Cost lower than 1000 Euro.
39.	Poppy [126]	2012	HBWR	Robot humanoid kit Interactive robot Open-source license.
40.	Lim andYeap [127]	2012	HBWR	6 DOFs walking robot.
41.	RQ-HUNO [128]	2014	HBWR	Robot humanoid kit.
42.	Red-Dragon V3 [129]	2014	HBWR	Mobile device-controlled robot.
43.	w00dBob [130]	2014	HBWR	A biped wooden robot controlled by Arduino Nano.
**Synthetic bipedal walking robots**				
44.	RotoFoot * [131,132,133,134]	2014	SBWR	Walking robot with rotary feet.
45.	Slider [135,136]	2018	SBWR	Walking robot without knees.
46.	LEO [137,138]	2021	SBWR	Multimodal walking robot with the possibility to fly as a drone.

* Name of robot proposed for use in this paper.

**Table 2:** Comparison of known modern bipedal robots based on human or bird’s walking.

Robot Year	Manufacturer	Height cm Mass, kg	Elements of the Control System					Type ** Number of Drives	Speed km/h Load, kg
			Joints	IMU	LIDAR	Camera	F/T		
**Walking robots based on human gait**									
HRP-5P 2013	Japan	183 101	Position	x	x	Stereo vision	4x	E. HD 37	- 13
Valkyrie 2013	NASA	187 129	Position Torque	7x		Multiple. cameras	2x	E. SEA 44	- -
Toro 2014	GAC Germany	174 76	Position Torque	2x		RGB&D camera		E. HD 39	1.8 10
Atlas–N. G. 2015	Boston Dynamics	150 75	Position Force		x	Stereo vision		H. S-v. 30	5.4
WALK-MAN 2015 (2018)	IIT Italy	191/185 132/102	Position Torque	2x		Multiple cameras	2x	E. SEA 29	- 10
Kengoro 2016	Tokyo University	167 56.9	Position Tension	x		Stereo vision	2x	E. Muscle Tend./106	- -
NimbRo-OP2 2017	Bonn University	135 19	Position	x		Stereo vision		E. DCSM 34	- -
TALOS 2017	PAL Spain	175 95	Position Torque	x		RGB&D camera		E. HD 32	- -
**Walking robot based on birds gait**									
Cassie * 2016	Agility Robotics	115 31	Position	x				E. CD 10	5
Digit 2019	Agility Robotics	155 42.2	Position	x	x	4x Depth camera		E. CD 16	- -

* Robot with only lower body. ** Drive type: E Electric, CD—Cycloid Drive, DCSM—DC servo Motors, HD—Harmonic Drive, SEA—Series Elastic Actuator., H.—Hydraulic, S-V.—Servo-Valves.

## Data Availability

Not applicable.

## Abbreviations

AI	artificial intelligence
ASD	autism spectrum disorder
BBWR	bird biped walking robot
BCI	brain-computer interface
BLDC	brushless DC
COG	centre of gravity
COT	cost of transport
CPG	central pattern generator
D_C_	central drive of legs move
D_G_	independent balancing drive
D_L_	drive of left foot
D_R_	drive of right foot
DC	direct current
DOFs	degrees of freedom
DSP	double support phase
EHA	electro-hydrostatic actuator
HBWR	human biped walking robots
ICT	information and communications technology
IMU	inertial measurement unit
LIDAR	light detection and ranging
PID	proportional–integral–derivative
SBWR	synthetic biped walking robots
SMA	shape memory alloys
SR	specific resistance
SSP	single support phase
VO	visual odometry
ZMP	zero moment point
